# Targeting de novo lipogenesis to mitigate kidney disease

**DOI:** 10.1172/JCI178125

**Published:** 2024-02-15

**Authors:** Haikuo Li, Benjamin D. Humphreys

**Affiliations:** 1 Division of Nephrology, Department of Medicine, and; 2 Department of Developmental Biology, Washington University in St. Louis, St. Louis, Missouri, USA.

## Abstract

Ten percent of the population worldwide suffers from chronic kidney disease (CKD), but the mechanisms driving CKD pathology are incompletely understood. While dysregulated lipid metabolism is one hallmark of CKD, the pathogenesis of cellular lipid accumulation remains unclear. In this issue of the *JCI*, Mukhi et al. Identify acyl-CoA synthetase short-chain family 2 (*ACSS2*) as a disease risk gene and demonstrate a role for *ACSS2* in de novo lipogenesis (DNL). Notably, genetic or pharmacological inhibition of DNL protected against kidney disease progression in mice. These findings warrant evaluation of DNL inhibition with respect to efficacy and safety in people with CKD.

## Identification of *ACSS2* with computational genomics

**Figure 1:**
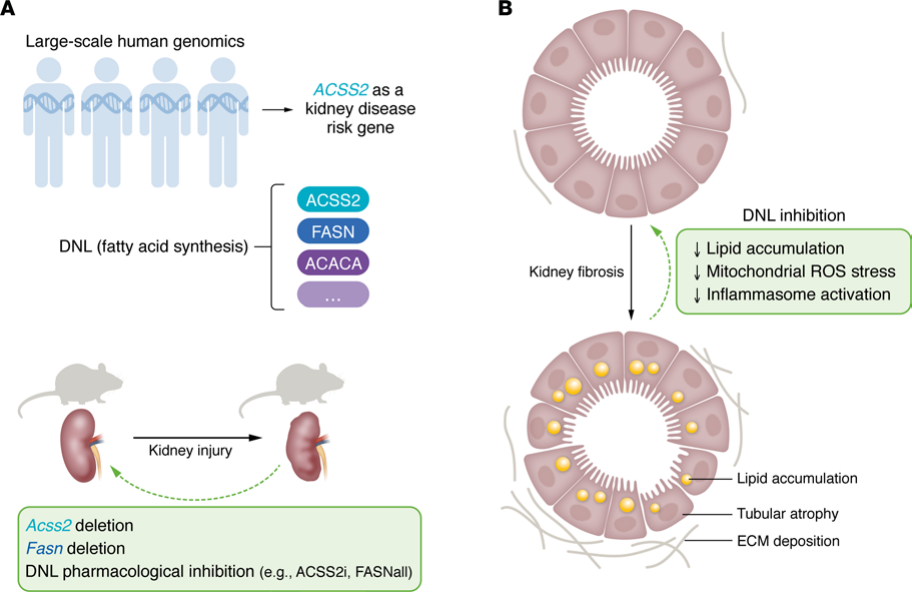
*ACSS2* has a role in DNL and is implicated in kidney fibrosis. (**A**) *ACSS2* was identified as a kidney disease risk gene in large-scale human genomics analysis. *ACSS2*, as well as other genes including *FASN* and *ACACA*, are involved in DNL. A protective role of DNL inhibition through either genetic deletion of *Acss2*, genetic deletion of *Fasn*, or pharmacological inhibition of DNL was observed in mouse models of kidney disease. (**B**) DNL inhibition results in a reduction of lipid accumulation, mitochondrial ROS, and inflammasome activation in tubular epithelial cells, which improves kidney function after injury. ECM, extracellular matrix.

Chronic kidney disease (CKD) is a major global health burden, resulting in 1.2 million deaths and 28.0 million years of life lost annually (1, 2). Liu et al. (3) previously performed a large-scale human GWAS and presented hundreds of genomic loci with strong association with kidney function indicators such as estimated glomerular filtration rate. In this issue of the *JCI*, Mukhi and authors first integrated orthogonal data sets, including expression and methylation quantitative trait loci and a single-nucleus assay for transposase accessible chromatin sequencing, to functionally annotate the GWAS results (4). With genetic mapping and statistical analysis, the authors prioritized several genes in close proximity on chromosome 20, including acyl-CoA synthetase short-chain family 2 (*ACSS2*), as potential kidney disease driver genes (Figure 1A) (4).

## *ACSS2* deletion protects against kidney disease

Mukhi and authors leveraged existing single-cell transcriptomics data, ISH, and immunostaining experiments to show that ACSS2 expression was highly specific to proximal tubule (PT) cells in human and mouse kidneys. Next, to study the functional importance of ACSS2, they generated global *Acss2*–knockout mice (*Acss2^–/–^*) with the CRISPR/Cas9 system. In multiple mouse models of kidney disease, including adenine-induced nephropathy and unilateral ureteral obstruction (UUO), *Acss2^–/–^* mice exhibited improved kidney function compared with control mice (Figure 1A), as reflected by reduced serum creatinine and blood urea nitrogen levels, improved tissue histology, and decreased expression of fibrosis markers such as fibronectin and α-smooth muscle actin. Of note, decreased ACSS2 expression was also observed in these bulk-level analyses, likely due to loss of PT cells upon kidney injury. A protective role for *Acss2* deletion was also validated with in vitro experiments on primarily isolated tubular epithelial cells, in which TGF-β1–induced fibrotic marker expression could be reversed in *Acss2^–/–^* cells.

ACSS2 catalyzes the activation of acetate into acetyl-CoA, which is subsequently used for fatty acid oxidation (FAO), cholesterol biosynthesis, fatty acid biosynthesis (i.e., de novo lipogenesis [DNL]), and histone posttranslational modifications (5, 6), but a role in CKD pathogenesis has not been described. To understand how this enzyme might affect kidney disease progression, the authors comprehensively analyzed the expression of marker genes for each pathway in *Acss2^–/–^* mice using a mouse model of kidney fibrosis. There were no substantial changes in histone acetylation, FAO, or cholesterol biosynthesis; however, genes involved in DNL, such as *Fasn* (encoding fatty acid synthase), *Acaca* (encoding acetyl CoA carboxylase), and upstream regulators *Srebp1* and *Scap*, were downregulated in *Acss2^–/–^* mice compared with wild-type mice. A reduction of DNL was also validated by a deuterated palmitate labeling experiment and Oil Red O staining, which suggested reduced lipid deposition in the kidney of *Acss2^–/–^* mice with UUO injury. The authors also generated tubule-specific *Fasn*-knockout mice and observed improved kidney function after kidney injury. These investigations identify a role for ACSS2 in DNL and suggest that perturbation of DNL genes in kidney disease is protective.

The kidney is a highly metabolically active organ, and PTs use lipids as the primary fuel source for mitochondrial oxidative phosphorylation and energy generation (7). Lipid accumulation in PT cells has been a well-known characteristic of CKD, leading to intracellular lipotoxicity and exacerbated tubular injury (8, 9). However, the mechanisms of lipid accumulation remain unclear. The increased kidney lipid accumulation has in the past been interpreted to reflect either compromised FAO (i.e., reduced lipid consumption) (10, 11) or increased lipid intake through fatty acid transporters such as CD36 and FATP2 (12, 13). The finding in Mukhi et al. that DNL provided another source of lipid accumulation in kidney disease is therefore important. We and others (14, 15) recently described perilipin 2 (PLIN2) as a marker of lipid droplets in tubular epithelial cells during kidney injury, and Mukhi et al. confirmed an upregulation of *Plin2* after kidney injury in their mouse models, as well as a decrease in *Plin2* expression after ACSS2 loss, further supporting the idea that inhibition of DNL can reduce lipid accumulation in kidney disease (4).

## DNL as a therapeutic strategy

To evaluate DNL targeting as a potential therapeutic approach, the authors treated wild-type mice with pharmacological inhibitors of FASN or ACSS2 in the context of kidney injury (Figure 1A). Treatment with FASNall, a selective FASN inhibitor (16), decreased fibrotic gene expression, improved tissue histology, and resulted in less lipid deposition after UUO surgery compared with controls. Administration of TVB-3664, another FASN inhibitor, also ameliorated the disease responses in vitro. The authors also demonstrated that targeting ACSS2 with ACSS2i, a small-molecule inhibitor, could protect mice from UUO-induced kidney fibrosis.

Next, the authors investigated the nephroprotective mechanisms of DNL inhibition. Using primary isolated tubule cells, they identified NADPH/NADP^+^ and GSH/GSSH (oxidized to reduced glutathione) ratios after TGF-β1 treatment in wild-type cells. These ratios were lower in *Acss2^–/–^* cells or cells treated with FASNall, indicating reduced oxidative damage with DNL inhibition. This finding was also supported by analysis of mitochondrial damage indicators (e.g., mitochondrial superoxide levels and membrane potential), which revealed reduced mitochondrial ROS accumulation after DNL inhibition. To study whether the mitochondrial defect was accompanied by inflammasome activation and pyroptosis, the authors measured gene expression in the NLRP3 inflammasome pathway, including NLRP3, CASP1, and GSDMD. These genes were upregulated after adenine- or UUO-induced mouse kidney injury, but DNL inhibition (through ACSS2i treatment, genetic *Acss2* knockout, or tubule-specific *Fasn* knockout) reduced this upregulation, suggesting reduced inflammasome activation and pyroptosis signaling. Collectively, these observations provide convincing evidence that pharmacological inhibition of DNL ameliorated kidney disease in mouse models through mechanisms linking DNL and kidney fibrosis through mitochondrial ROS and NLRP3 inflammasome activation (Figure 1B).

## Conclusions and future directions

Using large-scale human genetics and genomics analysis, this work identifies *ACSS2* as a kidney disease risk gene. Through comprehensive in vitro and in vivo experiments, the authors demonstrate the role of ACSS2 in DNL and the association of DNL with lipid accumulation and kidney fibrosis. In addition, inhibition of DNL effectively mitigated disease progression and protected against mitochondrial defects and inflammasome hyperactivation in kidney tubular cells.

Several questions remain. (a) The genomic locus prioritized in the computational genomics analysis is a gene-dense region containing multiple genes besides *ACSS2*. Whether other genes in this region are also disease-causing genes and how this locus is epigenetically regulated in disease are unknown. (b) It remains unclear whether ACSS2 is exclusively responsible for fatty acid synthesis in kidney or also involved in other related pathways. For example, genes associated with cholesterol biosynthesis, such as *Hmgcs1* and *Hmgcr*, in the *Acss2^–/–^* mice showed lower expression than in wild-type mice, although the variation was less pronounced compared with DNL genes. (c) The importance of DNL will need to be carefully evaluated in human CKD, and further experiments will be required to validate the effectiveness and safety of DNL inhibition in humans.

## Version 1 02/15/2024

Electronic publication
